# What Do We Know About *Staphylococcus aureus* and Oxidative Stress? Resistance, Virulence, New Targets, and Therapeutic Alternatives

**DOI:** 10.3390/toxics13050390

**Published:** 2025-05-13

**Authors:** Mírian Letícia Carmo Bastos, Gleison Gonçalves Ferreira, Isis de Oliveira Kosmiscky, Ieda Maria Louzada Guedes, José Augusto Pereira Carneiro Muniz, Liliane Almeida Carneiro, Ísis Lins de Carvalho Peralta, Marcia Nazaré Miranda Bahia, Cintya de Oliveira Souza, Maria Fâni Dolabela

**Affiliations:** 1 Biodiversity and Biotechnology Bionorte Network, Federal University of Para, Belém 66075-110, PA, Brazil; mirian.c.bastos@hotmail.com; 2 Postgraduate Program in Pharmaceutical Sciences, Federal University of Para, Belém 66075-110, PA, Brazil; gleison.ferreira@ics.ufpa.br (G.G.F.); isis.kosmiscky@ics.ufpa.br (I.d.O.K.); 3 Center for Biological Sciences, Federal University of Para, Belém 66075-110, PA, Brazil; iedaguedes@ufpa.br; 4 Brazilian National Primate Center, Ananindeua 67030-970, PA, Brazil; japcmuniz@gmail.com (J.A.P.C.M.); liliane.carneiro@cenp.gov.br (L.A.C.); 5 Bacteriology Section, Evandro Chagas Institute, Ananindeua 67030-000, PA, Brazil; lins.peralta@gmail.com (Í.L.d.C.P.); marciabahia@iec.gov.br (M.N.M.B.); cintyaoliveira@iec.gov.br (C.d.O.S.)

**Keywords:** *Staphylococcus aureus*, oxidative stress, antimicrobial resistance, biofilm, reactive oxygen species, alternative therapies

## Abstract

*Staphylococcus aureus* is associated with human infections, being a resistant bacterium involved in serious infections, and its virulence and resistance are linked to oxidative stress. In this study, we review the role of oxidative stress in the pathogenesis of this bacterium and its influence on immune system evasion, antibiotic resistance, and pharmacological targeting. *S. aureus* infection generates an intense inflammatory response in the host, evidenced by the activation of pro-inflammatory pathways, the exacerbated production of reactive oxygen species (ROS), and cellular oxidative stress. However, the bacterium develops protective mechanisms against damage, including the production of endogenous antioxidants, the formation of biofilms, and the regulation of redox metabolism, favoring pathogenicity and drug resistance. Resistance seems to be related to alterations in redox metabolism, which influences the sensitization of the immune system. Modulation of the redox response has emerged as a promising approach for developing new antibiotics and formulating more effective combination therapies to combat resistant infections. Natural compounds, including flavonoids, terpenes, and quinones, have demonstrated antibacterial properties by inducing oxidative stress in *S. aureus*. In summary, the involvement of oxidative stress is complex, with an increase in ROS in the infection and a reduction in immune system evasion and resistance, which could be an interesting therapeutic target.

## 1. Introduction

*Staphylococcus aureus* is a bacterium commonly found on the skin and in the nasal passages of healthy individuals. While often harmless, it can also cause a wide range of severe or even fatal infections [1]. This bacterium is responsible for various diseases, ranging from localized skin abscesses to systemic and chronic infections such as septicemia, endocarditis, pneumonia, and osteomyelitis [2]. Notably, *S. aureus* can colonize almost any human organ, sometimes leading to critical clinical conditions [3].

It is imperative to emphasize that *S. aureus* is equipped with numerous defensive mechanisms to evade the host immune system. A pivotal role in this process is fulfilled by protein A, which binds to the Fc region of antibodies, thus impeding the immune system’s capacity to effectively target and eradicate the bacterium [4]. *S. aureus* produces hemolysins, leukocidins, and exfoliative toxins, which induce tissue damage and facilitate bacterial colonization and persistence within the host [5]. In addition, enzymes such as coagulase and staphylokinase contribute to tissue invasion by promoting either the formation or degradation of fibrin clots, thereby aiding bacterial dissemination and evasion of the immune system [6].

Due to the indiscriminate use of antimicrobials, *S. aureus* has developed resistance to many routinely used antibiotics, including penicillin, and its resistance mechanism is mediated by the production of the β-lactamase enzyme. In addition, methicillin-resistant *Staphylococcus aureus* (MRSA) strains have emerged, and they are resistant to all penicillin, including methicillin, and almost all beta-lactam antibiotics, posing a significant challenge to clinical treatment [7]. Numerous infections result from antibiotic-resistant bacteria, commonly acquired in hospitals [8]. The treatment of these infections is further complicated by the emergence of MRSA and vancomycin-resistant *Staphylococcus aureus* (VRSA) strains, some of which have also developed multidrug resistance (MDR), posing significant challenges to clinical management [9,10].

Conversely, the discovery of new drugs capable of targeting resistant bacterial strains remains a significant challenge, with only two new antimicrobial agents introduced in recent decades: linezolid (2000) and daptomycin (2003) [11]. This raises concerns about a potential future scenario in which available antibiotics may become ineffective against human pathogens, leading to increased morbidity and mortality rates [12].

Antimicrobial agents exert their effects through various bacterial signaling pathways, producing bacteriostatic effects when targeting the 30S and 50S ribosomal subunits [13]. Bactericidal agents, however, interfere with protein translation [14], cell wall synthesis, and DNA replication and repair [15]. More recently, oxidative stress has been documented as a key mechanism of action for most bactericidal antibiotics [16,17], highlighting the need to further investigate the contribution of oxidative stress to the antibacterial effect of these agents.

In this study, we review the role of oxidative stress in the pathogenesis of this bacterium, its influence on immune system evasion, antibiotic resistance, and its potential as a pharmacological target. *S. aureus* infection triggers a robust inflammatory response in the host, characterized by the activation of pro-inflammatory pathways, the excessive production of reactive oxygen species (ROS), and cellular oxidative stress.

## 2. Results and Discussion

### 2.1. Oxidative Stress and Its Involvement in the Virulence and Resistance of S. aureus

The capacity of *S. aureus* to induce infections is associated with various virulence factors (Table 1). These factors facilitate the bacterium’s adhesion to surfaces, enable its evasion of the immune system, and induce cytotoxic damage to the host [6]. Virulence factors include the formation of bacterial biofilms and the production of hemolysins and staphyloxanthin, as well as the secretion of proteolytic enzymes, such as proteases, which contribute to tissue invasion and the progression of the infection [5,6].

The cell wall of *S. aureus* contains two components, peptidoglycans and lipoteichoic acids, which interact with Toll-like receptor 2 (TLR2) to trigger an inflammatory signaling cascade. This cascade involves the activation of intracellular pathways, including Nuclear Factor Kappa B (NF-κB) and Mitogen-Activated Protein Kinase (MAPK), leading to the production of pro-inflammatory cytokines and the induction of oxidative stress [18,19,20,21]. Furthermore, the activation of TLR2 has been demonstrated to be associated with an increase in caspase activity, which contributes to the process of cell apoptosis [22].

The nuclear factor erythroid 2-related factor 2 (NRF2) pathway is vital for nuclear transcription and the activation of antioxidant genes in response to external stimulation during inflammatory processes like mastitis. The activation of NRF2 is fundamental in reducing the damage induced by oxidative stress and exacerbated inflammation, and it is a relevant target for therapeutic strategies aimed at modulating the inflammatory response associated with oxidative stress [23].

Under physiological conditions, NRF2 is found in the cytosol bound to kelch-like enoyl-coenzyme A hydratase-associated protein 1 (KEAP1). Under oxidative stress conditions, Keap1 undergoes oxidation, thereby promoting the release and translocation of NRF2 to the nucleus. This process results in the activation of antioxidant genes, including heme oxygenase 1 (HO-1) and NAD(P)H: quinone oxidoreductase 1 (NQO1). These molecules are of significance in the neutralization of ROS and the protection of cells against oxidative damage [24].

*S. aureus* infection has been demonstrated to induce oxidative stress and activate the NRF2 pathway [25,26,27], in conjunction with an excessive increase in the production of inflammatory mediators. This has been shown to trigger irreversible cell damage, such as increased mitochondrial membrane permeability. The process releases cytochrome-c into the cytoplasm and subsequently activates caspase-associated apoptotic proteins. This series of events leads to the fragmentation of DNA and cell death [28]. Therefore, it can be posited that oxidative damage to cells may contribute to the progression of infectious inflammatory diseases [29] and apoptosis induced by oxidative stress [30].

In addition to its capacity to inflict harm upon the host, *S. aureus* has been shown to elicit an oxidative stress response, characterized by an augmentation in lipidic peroxidation levels and a concomitant diminution in the expression of antioxidant enzymes [31,32]. The protective mechanism is orchestrated by antioxidant enzymes (AOEs), including superoxide dismutase (SOD), catalase (CAT), and glutathione peroxidase (GPx), along with other antioxidant pathways that contribute to its survival [33].

The host’s immune response has been shown to contribute to the intensification of oxidative stress during the infectious process. This is because of the production of high concentrations of ROS and reactive chlorine species (RCS), including hypochlorous acid (HOCl), by activated macrophages and neutrophils as part of the antimicrobial defense response [34,35,36,37].

Oxidative stress can be exacerbated because the production of ROS and HOCl by the immune system leads to the oxidation of biomolecules, including amino acids, unsaturated fatty acids, carbohydrates, and nucleotides, resulting in the formation of electrophilic reactive species (RES) with electron-deficient centers. These molecules act as secondary reactive metabolites. Examples of such molecules include quinones, epoxides, and highly toxic dicarbonyl compounds, such as glyoxal and methylglyoxal [34,35,36,37,38].

However, the bacterium possesses antioxidant systems that facilitate its survival and pathogenicity, as well as low-molecular-weight thiols to attenuate oxidative stress [38]. In particular, the weight of thiol bacillithiol (BSH) has been identified as essential for the virulence of *S. aureus*, especially in macrophage-mediated infection trials [39,40].

The pathogenicity of *S. aureus* is also influenced by the concentration of oxygen in the infectious environment. Changes in the levels of this nutrient can modulate the expression of virulence factors, triggering processes such as adherence, invasiveness, and persistence in host cells. These processes are fundamental to the colonization of cell surfaces and tissues [41,42,43,44,45].

In this regard, it can be observed that the bacterium exhibits the capacity to adapt to variations in the concentration of oxygen in the environment. It does this by activating redox-dependent processes that regulate the expression of essential genes in the transition from aerobic to anaerobic growth. Thiol-specific redox systems have been shown to play a role in protecting cells against toxic oxygen species, as well as maintaining the intracellular thiol–disulfide balance and supplying reducing power for important reductive enzymes, such as ribonucleotide reductases, which are involved in DNA synthesis and repair [46].

It is evident that the interactions between *S. aureus* and the host result in the generation of various reactive species, necessitating highly efficient systems by the bacteria to ensure successful infection, dissemination, and survival. Among the defense mechanisms are redox-sensing regulators, such as staphylococcal accessory regulator Z (SarZ), multiple gene regulator A (MgrA), hydrogen peroxide regulator (HypR), and quinone-sensing regulator (QsrR), which utilize conserved cysteine (Cys) residues to detect and respond to ROS, RCS, and RES through post-translational thiol modifications, leading to the activation of their specific Cymtory operons (regulons) in *S. aureus* (Table 2 [47,48,49]).

The interconnection between oxidative stress regulatory pathways in *Staphylococcus aureus* is a complex and highly coordinated process, involving the integration of multiple detection and response systems to ROS, RCS, and RES. One of the central pathways is the NRF2 pathway, which plays a crucial role in activating antioxidant genes in response to oxidative stress [23,24,25,26,27]. Under stress conditions, NRF2 dissociates from the KEAP1 complex and translocates to the nucleus, where it activates the expression of enzymes such as HO-1 and NAD(P)H: NQO1, which neutralize ROS and protect cells from oxidative damage [24]. This pathway is interconnected with other regulatory pathways, such as HypR and QsrR, which detect and respond to different types of oxidative stress [47,48,49].

The interaction between these pathways occurs through post-translational modifications, such as the thiolation of cysteine residues, which act as redox sensors [47,48,49]. For example, HypR and QsrR contain conserved cysteine residues that are modified in response to oxidative stress, leading to the activation of their regulatory functions [50]. These modifications allow the pathways to communicate and coordinate an integrated response to stress. Additionally, the NRF2 pathway can modulate the expression of genes that influence the activity of HypR and QsrR, creating a regulatory network that amplifies the antioxidant response [23,24,25,26,27]. For instance, NRF2 activation can increase the production of enzymes that reduce H_2_O_2_ levels, alleviating the burden on HypR [50].

Similarly, the detoxification of quinones mediated by QsrR can reduce the formation of RES, decreasing the need for NRF2 activation [47,48,49]. This interconnection between pathways ensures that *S. aureus* can adapt to different types of oxidative stress, maintaining redox homeostasis and promoting its survival in hostile environments, such as during host infection.

An instance of adaptation manifests during the process of growth under microaerophilic conditions, wherein *S. aureus* displays constitutive resistance to hydrogen peroxide (H_2_O_2_). This resistance remains unmodulated by prior exposure to low doses of the aforementioned agent, suggesting that the bacterium maintains a robust defense mechanism that is independent of external induction. This adaptation may be attributed to the overexpression of catalase KatA, an enzyme responsible for the degradation of H_2_O_2_, conferring protection to *S. aureus* against oxidative damage and facilitating its survival in hostile environments. This oxidative stress resistance enhances the bacterium’s ability to persist within the host and contributes to its increased pathogenicity [50].

Furthermore, Cys-containing molecules, including thioredoxin and glutathione, have been demonstrated to play a pivotal role in protecting cells against oxidative stress [51,52]. In the context of Gram-positive bacteria, regulatory thiols such as mycothiol, coenzyme A, and bacillithiol have been postulated to function as antioxidants [53]. The regulation of cy metabolism by Cysteine Metabolism Regulator (CymR) also influences bacterial virulence and adaptation, acting to control the expression of genes involved in biofilm formation and the utilization of host sulfur sources, such as taurine and homocysteine [54]. The crystallography of CymR shows a dimeric structure in which each monomer has a DNA-binding domain; this allows for the regulation of gene expression, which favors virulence and the response to oxidative stress [55].

Research has demonstrated that protein S modulates redox modifications in *S. aureus* under conditions of oxidative stress through a process known as bacillithiolation. This process involves the modification of proteins by BSH, a mechanism that functions as a protective response against oxidative damage. BSH, a cofactor of thiol S-transferases, plays a pivotal role in the detoxification of reactive oxygen species, the maintenance of metal homeostasis, and the regulation of virulence. Furthermore, BSH has been implicated in resistance to antibiotics, including fosfomycin [56]. BSH participates in traditional processes under the effect of HOCl-mediated stress, where the S-bacilitiolated proteins most inactivated in this process in *S. aureus* are glycolytic glyceraldehyde-3-phosphate dehydrogenase (GapDH) and aldehyde dehydrogenase (AldA) [57,58,59,60].

In the context of redox balance, a further system of paramount importance to the redox homeostasis of *S. aureus* is thioredoxin (Trx). This system is catalyzed by thioredoxin reductase (TrxR), which facilitates the maintenance of proteins in their reduced state, thereby enabling thiol-disulfide exchange reactions. This process serves to regulate protein function and protect against oxidative stress by reducing intracellular ROS levels [61].

The importance of Trx for adaptation to oxidative stress is evidenced by the positive regulation of the TrxA and TrxB genes after exposure to oxidizing agents such as menadione and hydroperoxide, suggesting that these genes play a vital role in the bacteria’s response to oxidative stress. Furthermore, TrxB is essential for the survival of *S. aureus* in adverse environments and is a promising target for studies on new antimicrobials since there is a difference between this redox system and those of mammals [62].

The yjbH gene plays an important role in resistance to oxidative stress. It has been shown to modulate the bacterial response to free radicals, thereby helping to protect the cell against damage caused by peroxides and other oxidizing agents. Deletion of the *yjbH* gene has been associated with pathogenicity in *Staphylococcus aureus*, and it has been shown to affect the survival of mice in experimental models [63,64].

The virulence of *S. aureus* is also influenced by operons that regulate immune system evasion mechanisms and drug resistance. In methylglycine-resistant *S. aureus* (MRSA) strains, for example, staphyloxanthin is mediated by the staphyloxanthin biosynthesis operon (or staphyloxanthin biosynthesis operon), which includes genes such as svnA, svnB, and svnC. The system under discussion is related to the neutralization of ROS produced by cells of the immune system, such as neutrophils and macrophages [64]. Another operon is msaABCR, the function of which is to maintain membrane integrity during oxidative stress by cross-linking peptidoglycan. Both processes favor the perpetuation of the infection [65,66,67,68].

Biofilm production is among the most significant virulence factors in the colonization and evasion of antibiotics produced by bacteria. It occurs in response to environmental and physiological pressures, thereby allowing *S. aureus* to establish persistent infections that are highly resistant to conventional therapies. The process under discussion is one that involves the biopolymerization of exopolysaccharides, as well as extracellular DNA and proteins. The purpose of this process is to protect the bacteria in question [69].

In addition to the structural role of the biofilm matrix, studies suggest that *S. aureus* biofilm cells display enhanced oxidative stress responses when compared to their planktonic counterparts. Proteomic analyses have revealed the presence of antioxidant enzymes, including catalase and superoxide dismutase, within the biofilm matrix, suggesting an active mechanism for detoxifying ROS and protecting the cells from oxidative damage [70,71,72,73].

Furthermore, elevated levels of stress-response proteins—such as oxidoreductases, reductases, and glyceraldehyde-3-phosphate dehydrogenase (GapDH)—have been observed in biofilm cells, indicating an upregulation of redox homeostasis pathways [70,71]. Although ROS levels were not directly quantified in these studies, the findings indicate that biofilm cells possess a more robust antioxidant defense system, which may contribute to increased resistance to antibiotics and immune clearance [70,71,72,73].

The synergistic action of substances is a very promising therapeutic alternative, since synergism with natural antioxidants can enhance antimicrobial action [74,75]. A recent study pointed to the synergistic action of quercetin with ciprofloxacin and gentamicin against a strain of *S. aureus*, where the association was able to promote an increase in the activity of these two drugs, and the inhibition of the biofilm [74,76]. Another study demonstrated the synergistic activity of ascorbic acid with rifampicin and vancomycin against strains of methicillin-resistant MRSA, where all MICs were reduced by this combination [77,78]. These findings support the potential of antioxidant strategies as adjunctive therapies in clinical settings to improve antibiotic efficacy and reduce side effects [79,80,81].

In the regulatory process, the glucose-inhibited division protein A (GbaA) gene, belonging to the tetracycline repressor family (TetR), can function as either a negative or positive regulator, depending on the bacterial species. In *S. aureus*, *GbaA* acts as a negative regulator by inhibiting signaling pathways that control the transcription of genes involved in biofilm formation. Additionally, it may be associated with the modulation of signaling pathways regulating the transcription of biofilm-related genes, such as those responsible for the production of poly-N-acetylglucosamine intercellular adhesin (PIA; Table 3), a gene transcript of the main components of the biofilm matrix in *S. aureus* [70].

Among the regulatory strategies that are not directly regulatory in nature that have been developed to modulate the response of *S aureus* to environmental stress is the post-transcriptional modification of ribosomal ribonucleic acid (rRNA) by methylation, a process that influences protein synthesis and antimicrobial resistance [71]. Kasugamycin resistance A (KsgA) is a methyltransferase responsible for the modification of an rRNA, specifically by catalyzing the methylation of adenine in the *16S* rRNA [72]. KsgA may influence the stress response indirectly by maintaining the efficiency of the translation of the proteins involved in eliminating ROS or repairing damage caused by oxidation. Furthermore, in some cases, the regulation of KsgA may affect the production of proteins that help protect the cell against oxidative stress, such as heat shock proteins (HSPs) or those involved in peroxide detoxification, although the specific interactions in *S. aureus* still require further investigation [73].

The enzymes involved in methionine maintenance seem to play a key role in surviving oxidative stress. In a study in which the Methionine Sulfoxide Reductase A1 (MsrA1), Methionine Sulfoxide Reductase A2 (MsrA2), Methionine Sulfoxide Reductase A3 (MsrA3), and Methionine Sulfoxide Reductase B (MsrB) genes were manipulated, it was observed that the deletion of the MsrA1 gene increased the sensitivity of *S. aureus* to oxidative stress, along with a reduction in virulence factors such as pigment production and adhesion. Conversely, *S. aureus* strains deficient in MsrB exhibited increased resistance to oxidative stress and expressed higher levels of virulence factors. Alterations in the MsrA2 and MsrA3 genes did not show significant changes in the studied parameters. These findings suggest that MsrA1 and MsrB may regulate each other, maintaining a balance in the oxidative stress response and survival of *S. aureus* [82].

Antimicrobial resistance appears to be associated with alterations in redox metabolism that influence immune sensitization. Infections with vancomycin-sensitive and vancomycin-resistant strains of *S. aureus* have been shown to increase ROS production. This increase can lead to cell damage, particularly in lymphocytes [81]. In addition, ciprofloxacin-resistant strains of *S. aureus* have been shown to be less sensitive to ROS, particularly superoxide anion (O_2_^−^), which interferes with the immune response and the efficacy of this drug. The mechanism of action of ciprofloxacin involves oxidative stress, as it disrupts cellular processes, unbalancing the production and detoxification of ROS, ultimately contributing to bacterial cell damage and death [82].

### 2.2. Possible Therapeutic Alternatives to Reduce the Impact of Oxidative Stress on S. aureus

The indiscriminate use of antibiotics to treat diseases caused by pathogenic bacteria can significantly exacerbate the problem of antimicrobial resistance and consequently limit the effectiveness of available drugs. When antibiotics are used inappropriately or excessively, they exert a strong selective pressure on bacterial populations. This favors the survival and proliferation of resistant strains that have mechanisms to neutralize or evade the action of the drugs [83]. Given this scenario, it is essential to look for other antimicrobial agents that can be derived from natural sources, such as plants, whose metabolites can act as pro-oxidants in resistant bacterial strains, interfering with resistance mechanisms and acting directly on the bacteria [80].

Metabolites of plant origin have been shown to possess both pro-oxidant and antioxidant properties [84,85,86,87,88,89,90,91,92,93]. The pro-oxidant effect has demonstrated significant promise in resistant bacterial strains, as it has been observed to interfere with resistance mechanisms and act directly on the bacteria [84]. If left unchecked, ROS are highly damaging to cells, as they directly and/or indirectly damage various cellular components. Thus, increasing oxidative stress has emerged as a promising approach, not only to eliminate pathogens but also to modulate virulence and the bacterial response to antimicrobial treatment [84]. The hypothesis is that the heme oxygenase (HO) generated by bactericidal antibiotics exerts antibacterial efficacy partly due to the lack of effective cellular detoxification mechanisms for HO• in bacteria [91,92]. Conversely, bacteriostatic agents reduce cellular respiration and do not produce HO• [87,94,95,96,97].

**Table 1:** Role of oxidative stress in *S. aureus* pathogenesis and protection.

Process	Role in Pathogenesis	Protective Mechanisms	References
Production of ROS/RCS by the immune system	Neutrophils and macrophages release ROS/RCS to eliminate *S. aureus*, causing oxidative damage	*S. aureus* has developed resistance to oxidative stress through antioxidant systems and immune evasion	[34,35,36,37]
TLR2 activation and inflammatory pathways	TLR2 activation triggers the production of pro-inflammatory cytokines, increasing oxidative stress	Modulation of TLR2 and inflammatory pathways can minimize host damage and improve bacterial persistence	[18,19,20,21]
Oxidative damage to host cells	Excess ROS can damage immune cells, favoring infection persistence	Bacterial survival may be enhanced by reducing immune system efficacy	[28,29,30]
Biofilm production as a resistance mechanism	Biofilm formation protects *S. aureus* against ROS and antibiotics, increasing resistance	Biofilm prevents ROS and antibiotic penetration, enhancing bacterial resilience	[69,70]
Bacterial antioxidant response (SOD, CAT, Gpx)	*S. aureus* uses antioxidant enzymes to detoxify ROS and survive oxidative stress	Antioxidant enzymes neutralize ROS before they cause cellular damage	[31,32,33]
Role of BSH in ROS defense	Bacillithiol protects bacterial proteins from oxidative damage and contributes to virulence	BSH functions as a protective system against reactive species, enhancing bacterial resistance	[39,40]
Resistance mediated by operons (staphyloxathin msaABCR)	Resistance genes such as *staphyloxanthin* neutralize ROS, while *msaABCR* maintains membrane integrity	These operons facilitate adaptation and resistance to oxidative stress in hostile environments	[64,65,66,67,68]
Redox metabolism regulation (CymR, Trx, KatA)	Regulators such as CymR and Trx aid in adaptation to oxidative environments, increasing bacterial survival	Redox metabolism is regulated to prevent cellular damage and maintain bacterial homeostasis	[46,47,48,49,50,51,52,53,54,55]
Post-transcriptional modification of rRNA (KsgA)	KsgA improves the efficiency of translating proteins protective against oxidative stress	Translation regulation protects essential proteins for stress response	[71,72,73]
Enzymes involved in methionine maintenance (MsrA1, MsrB)	MsrA1 and MsrB regulate oxidative stress response and influence bacterial virulence	Self-regulation of MsrA1 and MsrB ensures balance in oxidative stress response	[74]
Influence of antimicrobial resistance on redox metabolism	Antibiotic-resistant strains, such as those resistant to ciprofloxacin, exhibit lower sensitivity to oxidative stress	Antimicrobial resistance can increase tolerance to oxidative stress, reducing antibiotic effectiveness	[75,76]

ROS—reactive oxygen species; RCS—reactive chlorine species; RES—reactive electrophilic species; TLR2—Toll-like receptor 2; Nrf2—nuclear factor erythroid 2-related factor 2; Trx—thioredoxin; TrxR—thioredoxin reductase; KatA—catalase A; SOD—superoxide dismutase; Gpx—glutathione peroxidase; BSH—bacillithiol; CymR—Cysteine Metabolism Regulator; MgrA—multiple gene regulator A; HypR—Hypochlorite-Responsive Regulator; QsrR—quinone-sensing regulator R; GapDH—glyceraldehyde-3-phosphate dehydrogenase; AldA—aldehyde dehydrogenase A.

**Table 2:** Overview of the key regulatory mechanisms that *S. aureus* employs to manage oxidative stress, enhance virulence, and resist antimicrobial treatments.

Regulatory Mechanism	Description	Role in Oxidative Stress/Pathogenesis	References
TLR2 Activation	Toll-like receptor 2 (TLR2) activation triggers pro-inflammatory cytokine production and oxidative stress.	Induces oxidative stress and inflammation, contributing to host cell damage.	[18,19,20,21]
NRF2 Pathway	Nuclear factor erythroid 2-related factor 2 (NRF2) activates antioxidant genes in response to oxidative stress.	Protects host cells from oxidative damage by upregulating antioxidant enzymes like HO-1 and NQO1.	[23,24,25,26,27]
Antioxidant Enzymes (SOD, CAT, GPx)	Superoxide dismutase (SOD), catalase (CAT), and glutathione peroxidase (GPx) neutralize reactive oxygen species (ROS).	Protects *S. aureus* from oxidative damage, enhancing survival and pathogenicity.	[31,32,33]
Bacillithiol (BSH)	A low-molecular-weight thiol that protects bacterial proteins from oxidative damage.	Enhances bacterial resistance to oxidative stress and contributes to virulence.	[39,40]
Redox-Sensing Regulators (SarZ, MgrA, HypR, QsrR)	Regulators that detect ROS, reactive chlorine species (RCS), and reactive electrophilic species (RES) through thiol modifications.	Activates specific operons to protect *S. aureus* from oxidative stress and maintain redox balance.	[47,48,49]
Catalase KatA	An enzyme that degrades hydrogen peroxide (H_2_O_2_), providing resistance to oxidative stress.	Protects *S. aureus* from oxidative damage, especially under microaerophilic conditions.	[50]
Thioredoxin (Trx) System	Thioredoxin and thioredoxin reductase (TrxR) maintain proteins in their reduced state, protecting against oxidative stress.	Essential for bacterial survival in adverse environments and a potential target for new antimicrobials.	[61,62]
Cysteine Metabolism Regulator (CymR)	Regulates cysteine metabolism and biofilm formation, influencing bacterial virulence and adaptation to oxidative stress.	Controls sulfur source utilization and biofilm formation, enhancing bacterial survival.	[53,54,55]
Post-Transcriptional Modification (KsgA)	Methyltransferase that modifies rRNA, influencing protein synthesis and oxidative stress response.	Enhances the translation of proteins involved in oxidative stress resistance.	[71,72,73]
Methionine Sulfoxide Reductases (MsrA1, MsrB)	Enzymes involved in repairing oxidative damage to methionine residues.	Regulates oxidative stress response and virulence factors in *S. aureus*.	[74]
Staphyloxanthin Biosynthesis Operon	Operon involved in the production of staphyloxanthin, a carotenoid pigment that neutralizes ROS.	Protects *S. aureus* from oxidative stress, enhancing virulence and resistance to immune defenses.	[64,65]
msaABCR Operon	Operon that maintains membrane integrity during oxidative stress and regulates biofilm formation.	Enhances bacterial resistance to oxidative stress and promotes biofilm formation, aiding in persistent infections.	[66,67,68]
Glucose-Inhibited Division Protein A (GbaA)	A regulator that inhibits biofilm-related gene transcription in *S. aureus.*	Modulates biofilm formation, influencing bacterial resistance to oxidative stress and antibiotics.	[70]
SigB and GraRS Regulons	Regulons involved in the response to cell wall stress and general stress.	Protects *S. aureus* from oxidative stress and other environmental stresses.	[87]
PerR, HypR, QsrR, MhqR, CtsR, HrcA Regulons	Regulons involved in the oxidative stress response and protein damage repair.	Protects *S. aureus* from oxidative damage and maintains redox balance.	[87]

ROS: reactive oxygen species; RCS: reactive chlorine species; RES: reactive electrophilic species; TLR2: Toll-like receptor 2; NRF2: nuclear factor erythroid 2-related factor 2; SOD: superoxide dismutase; CAT: catalase; GPx: glutathione peroxidase; BSH: bacillithiol; Trx: thioredoxin; TrxR: thioredoxin reductase; CymR: Cysteine Metabolism Regulator; KsgA: Kasugamycin resistance A; MsrA1: Methionine Sulfoxide Reductase A1; MsrB: Methionine Sulfoxide Reductase B.

**Table 3:** Genes involved in biofilm formation in *S. aureus*.

Gene	Function	Role in Biofilm Formation
GbaA	TetR family regulator; acts as a negative regulator of biofilm-related gene transcription in *S. aureus*	Inhibits signaling pathways controlling the transcription of biofilm-related genes, modulating bacterial resistance
PIA	Main component of the biofilm matrix; contributes to *S. aureus* adhesion and biofilm resistance	Facilitates biofilm cohesion and structural stability, allowing increased bacterial persistence

PIA—Polysaccharide Intercellular Adhesin; TetR—tetracycline repressor family regulator.

Research into the natural active ingredients present in plants is a promising strategy for combating MRSA, which is often associated with the induction of oxidative stress as a mechanism of action. For instance, the monoterpene 1,8-cineole (eucalyptol) inhibited MRSA at low concentrations (7.23 mg/mL) and increased cell membrane permeability, causing the extravasation of nucleic acids and proteins compared to untreated strains. This effect is associated with lipid peroxidation, which further contributes to oxidative stress. The compound under investigation elicited an augmentation in the generation of ROS, reaching 17,462 fluorescence units in treated bacterial cells, concomitant with a diminution in the activities of antioxidant enzymes [96].

Other bioactive compounds, such as ginsenoside Rb1 (Rb1), extracted from *Panax ginseng*, have anti-inflammatory, antioxidant, and anti-tumor activities. In an acute lung injury (ALI) induction model, Rb1 reduced oxidative stress by suppressing the accumulation of malondialdehyde (MDA) and myeloperoxidase (MPO), as well as increasing the activities of the antioxidant enzymes superoxide dismutase 1 (SOD1), CAT, and glutathione peroxidase 1 (GPX1), strengthening the defense against oxidative stress. It also reduced perivascular edema and neutrophilic infiltration in the lungs of mice, increasing antioxidant defense by activating the NRF2 signaling pathway and improving hematological parameters. In addition, Rb1 therapy significantly reversed the messenger ribonucleic acid (mRNA) and protein expression of genes related to mitochondrial apoptosis, compared to an untreated ALI group, in vivo and in vitro [94].

A notable example is the flavonoid glabridin, isolated from *Glycyrrhiza glabra*, which exhibited antibacterial activity (a minimum inhibitory concentration (MIC) ranging from 3.12 to 25 μg/mL) against multidrug-resistant clinical isolates of *S. aureus*. It was observed that high concentrations of glabridin resulted in increased oxidative stress; however, when low concentrations of the flavonoid were utilized, the pharmacological effect was associated with a reduction in oxidative stress. Furthermore, glabridin exhibited a potentiated action in the presence of antibiotics, reducing the MIC of norfloxacin, oxacillin, and vancomycin by up to fourfold, while its own MIC was reduced by up to eightfold in the presence of these agents [95].

Oxidative stress and membrane permeability were explored as mechanisms underlying the antibacterial activity of an aqueous extract of *Syzygium aromaticum* seeds against *Staphylococcus aureus*. These seeds contained several bioactive compounds, including eugenol acetate, β-caryophyllene, eugenin, eugenol, and methyl salicylate. The extract exhibited strong antimicrobial activity, with a minimum inhibitory concentration (MIC) of 0.06 mg/mL and a minimum bactericidal concentration (MBC) of 0.10 mg/mL. Following exposure, *S. aureus* cells showed a significant increase in superoxide anion radicals, triggering an upregulation of antioxidant defenses such as superoxide dismutase and catalase. Concurrently, reduced glutathione levels and elevated malondialdehyde indicated oxidative stress and lipid peroxidation, confirming membrane damage as part of the extract’s bactericidal effect [98].

Lapachol exhibits antimicrobial activity against *S. aureus*, primarily by inducing oxidative stress. RNA-seq analysis revealed that lapachol treatment upregulated regulons associated with the oxidative stress response, including PerR, HypR, QsrR, MhqR, CtsR, and HrcA, as well as SigB and GraRS, which are linked to cell wall and general stress responses. This redox-based mechanism was supported by increased bacillithiol (BSH) redox potential, elevated endogenous ROS, accelerated H_2_O_2_ detoxification, and the in vivo thiol oxidation of GapDH and HypR. Catalase KatA and the Brx/BSH/YpdA pathway were identified as protective against ROS induced by lapachol. Notably, no evidence of protein alkylation or irreversible aggregation was observed. These findings suggest that lapachol’s antimicrobial activity in *S. aureus* is mediated by ROS generation and the disruption of redox homeostasis rather than by covalent protein modification [99].

**Figure 1:**
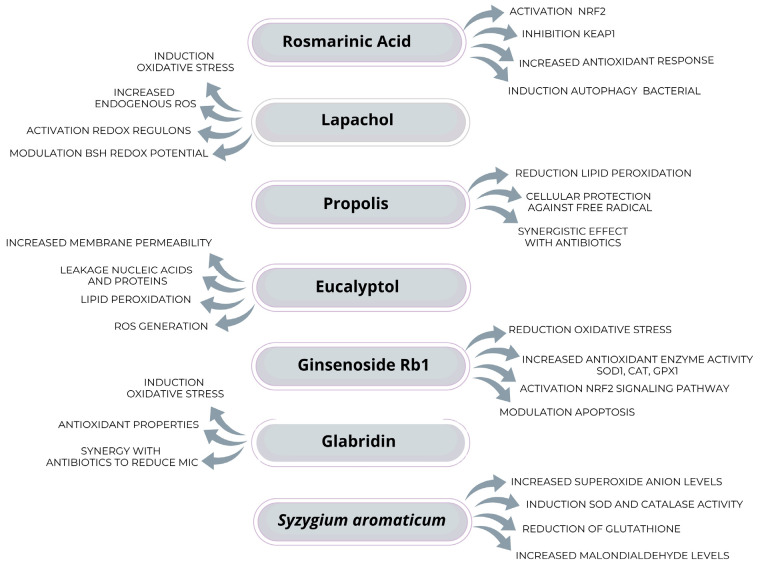
Oxidative stress modulation and antibacterial mechanisms of natural compounds against *S. aureus*. ROS—reactive oxygen species; NRF2—nuclear factor erythroid 2-related factor 2; KEAP1—kelch-like ECH-associated protein 1; BSH—bacillithiol; SOD—superoxide dismutase; CAT—catalase; GPX1—glutathione peroxidase 1; MIC—minimum inhibitory concentration.

In the search for effective therapies against bacterial pneumonia, rosmarinic acid (RA), a compound found in several medicinal plants, has been highlighted for its antioxidant and antimicrobial properties. RA’s mechanism of action involves the covalent inhibition of KEAP1, a protein responsible for recognizing and directing the transcription factor NRF2 for degradation by the proteasome. By inhibiting KEAP1, NRF2 accumulates in the cytosol and is subsequently translocated to the nucleus, where it activates genes involved in the antioxidant response, thereby enhancing cellular defense against oxidative stress. In addition, treatment with RA showed a bactericidal effect on macrophages, inducing the autophagic pathway, which promotes the accelerated eradication of bacteria and the resolution of inflammation. These effects contribute to recovery from the disease and highlight the therapeutic potential of RA in combating pneumonia caused by MRSA ([100], Figure 1).

In addition to novel natural products, the integration of oxidative stress modulation into clinical antibiotic strategies may offer complementary approaches to overcome bacterial resistance. Several studies have shown that bactericidal antibiotics (such as fluoroquinolones, aminoglycosides, and β-lactams) lead to the generation of reactive oxygen species (ROS) as part of their mechanism of action, contributing to bacterial cell death [101].

Although these classes act on different primary targets, they converge in the induction of oxidative stress. Specifically, fluoroquinolones, by inhibiting topoisomerase II, lead to the formation of DNA breaks, stimulating ROS production. Aminoglycosides have been observed to promote the mistranslation of proteins, thereby compromising cell membrane integrity and activating oxidative stress systems. Conversely, β-lactams, by interfering with cell wall synthesis, and daptomycin, by depolarizing the membrane, trigger cellular mechanisms that also lead to the formation of reactive species, potentiating the antibacterial effect of these drugs [101,102].

The combination of antibiotics with oxidative stress inducers, such as oxidizing agents, has been demonstrated to enhance the efficacy of antimicrobial treatment. For instance, the combination of antibiotics that generate oxygen radicals with oxidizing agents has been shown to potentiate the bactericidal effect, thereby enhancing the efficacy against bacterial biofilms that would otherwise exhibit significant resistance. This strategy is of particular relevance in clinical settings where antibiotic resistance is a growing concern [102].

## 3. Conclusions

*S. aureus* infection induces an intense inflammatory response in the host, as evidenced by the activation of pro-inflammatory pathways, increased ROS production, and cellular oxidative stress. A reduction in oxidative stress is involved in *S. aureus* virulence and resistance, influencing its ability to evade the immune system and altering its response to antimicrobial therapy. Conversely, bacteria have evolved defensive mechanisms to combat oxidative damage, encompassing the synthesis of endogenous antioxidants, the formation of biofilms, and the regulation of redox metabolism. These mechanisms not only promote pathogenicity but also enhance drug resistance.

## 4. Future Directions

There is a scarcity of clinical studies validating the proposed therapeutic alternatives, particularly concerning natural compounds. Despite the wealth of laboratory data and the promise demonstrated by flavonoids, terpenes, and quinones in inducing oxidative stress in *S. aureus*, the transition of these findings into practical applications remains uncertain and challenging. Most studies are confined to in vitro or animal models, with limited progress towards human trials. Furthermore, there is insufficient exploration of the toxicity, bioavailability, and drug interactions of these compounds in real clinical settings. For these alternatives to become viable, it is crucial to invest in translational research that integrates basic and applied knowledge, ensuring not only efficacy but also safety and feasibility for large-scale use. Without this, the therapeutic potential of these compounds may remain underutilized, perpetuating the antimicrobial resistance crisis.

## Abbreviations

The following abbreviations are used in this manuscript:

AldA	aldehyde dehydrogenase
ALI	acute lung injury
AOEs	antioxidant enzymes
BSH	bacillithiol
CAT	catalase
CymR	Cysteine Metabolism Regulator
Cys	cysteine
DNA	Desoxyribonucleic Acid
GbaA	glucose-inhibited division protein A
GapDH	glycolytic glyceraldehyde-3-phosphate dehydrogenase
GPx	glutathione peroxidase
GPX1	glutathione peroxidase 1
H_2_O_2_	hydrogen peroxide
HO	heme oxygenase or hydroxide
HO-1	heme oxygenase 1
HOCl	hypochlorous acid
HypR	hydrogen peroxide regulator
KEAP1	kelch-like enoyl-coenzyme A hydratase-associated protein 1
KatA	catalase A
Ksg	Kasugamycin
KsgA	Kasugamycin resistance A
MAKP	Mitogen-Activated Protein Kinase
MBC	minimum bactericidal concentration
MDA	malondialdehyde
MDR	developed multidrug resistance
MIC	minimum inhibitory concentration
MPO	myeloperoxidase
MgrA	multiple gene regulator A
mRNA	messenger ribonucleic acid
MRSA	methicillin-resistant *Staphylococcus aureus*
Msr	Methionine Sulfoxide Reductase
MrsA1	Methionine Sulfoxide Reductase A1
MrsA2	Methionine Sulfoxide Reductase A2
MrsA3	Methionine Sulfoxide Reductase A3
MrsB	Methionine Sulfoxide Reductase B
NADPH	Nicotinamide Adenine Dinucleotide Phosphate
NF-κB	Nuclear Factor Kappa B
NQO1	quinone oxidoreductase 1
NRF2	nuclear factor erythroid 2-related factor 2
O_2_^−^	superoxide anion
PIA	poly-N-acetylglucosamine intercellular adhesin
QsrR	quinone-sensing regulator
RA	rosmarinic acid
RES	electrophilic reactive species
RCS	reactive chlorine species
rRNA	ribosomal ribonucleic acid
ROS	reactive oxygen species
SOD	superoxide dismutase
SOD1	superoxide dismutase 1
SarZ	staphylococcal accessory regulator Z
TetR	tetracycline repressor family
regu2	Toll-like receptor 2
Trx	thioredoxin
TrxR	thioredoxin reductase
VRSA	vancomycin-resistant *Staphylococcus aureus*
